# Comparison between H-FABP & troponin i as outcome predictors in sepsis and sepsis-related LV dysfunction

**DOI:** 10.1186/2197-425X-3-S1-A753

**Published:** 2015-10-01

**Authors:** HM Sherif, M AbulWafa, A Gaber, W Sami

**Affiliations:** Faculty of Medicine, Cairo University, Critical Care Medicine, Cairo, Egypt

## Background

The emerging cardiac biomarker heart-type fatty acid-binding protein (H-FABP) is rapidly released from cardiomyocytes into the circulation shortly after the onset of the cell damage. Few studies have investigated its utility in critically ill patients and whether it offers a similar and even superior power to the conventional cardiac biomarkers.

## Objective

Estimation of the prognostic significance of H-FABP as an independent risk factor in patients with septic shock and the prevalence of sepsis related myocardial dysfunction, in comparison to Troponin I.

## Methods

Fifty ICU patients (pts.) with sepsis were enrolled in this study. All pts. were subjected to APACHE II score as a clinical scoring system on admission and every 24 h during the ICU stay. All pts. were also investigated for the serum levels of both H-FABP and Troponin I during the first 24 h after admission. Using modified Simpson´s method, echocardiographic left ventricular (LV) end-diastolic volume (LVEDV), LV end-systolic volume (LVESV) and LV % ejection fraction (%EF) were calculated on admission and after 24 h.

## Results

The pts. were divided into 12 pts. (mean: 50.2 ± 21 years) suffering from sepsis with stable hemodynamics (group-1), and 38 patients (mean: 58.4 ± 19.2 years) with septic shock (group-2). Compared to group-1, H-FABP of group-2 showed a significant higher values (76.3% vs. 33.3% of pts., *P* < 0.05), but the data was comparable for Troponin I. In both groups; compared to pts. with negative H-FABP, pts. with positive H-FABP showed a significant increased number of pts. (66% vs. 34%, *P* < 0.05), but the data was comparable for Troponin I. Compared to group-1, APACHE II score in group-2 showed significantly higher values (31.9 ± 9.3 vs.16.2 ± 7.1, *P* < 0.001). In both groups, the positive H-FABP pts. had significant higher APACHE II score than the negative H-FABP pts. (32.3 ± 8.7 vs. 20.1 ± 11 of pts., *P* < 0.001), but the data was comparable for Troponin I. Despite the positive H-FABP pts. and negative H-FABP pts. were comparable for LV%EF after 24 h of admission, but the positive H-FABP pts. showed significant increased LV volumes (LVEDV=105 vs. 77 ml, *P* < 0.05, and LVESV=49 vs. 33 ml, *P* < 0.05), respectively. The mortality rate was significantly higher in group-2 vs. group-1 (78.9% vs. 41.7%, *P* < 0.05). H-FABP was a better prognostic marker than Troponin I; it showed a higher prevalence of mortality (88% vs. 35%, *P* < 0.001) with good correlation (r = 0.54, *P* < 0.05). Multivariate regression analysis showed that the number of organ dysfunction with positive H-FABP pts. raised the odds of mortality 7.5 times (*P* < 0.05).

## Conclusions

H-FABP is a good prognostic marker and an independent risk factor for mortality in patients with severe sepsis and septic shock than Troponin I. During the first 24 hours of ICU admission, the positive H-FABP patients showed significant relation with sepsis-related LV systolic myocardial dysfunction.

